# New Risk Factor for SIDS? Peaks in Cot Deaths Associated with Heat Waves

**DOI:** 10.1289/ehp.123-A185

**Published:** 2015-07-01

**Authors:** Carrie Arnold

**Affiliations:** Carrie Arnold is a freelance science writer living in Virginia. Her work has appeared in *Scientific American*, *Discover*, *New Scientist*, *Smithsonian*, and more.

It’s every parent’s worst nightmare: A healthy infant goes down for a nap but never wakes up. Researchers have begun to identify a number of risk factors for sudden infant death syndrome (SIDS, also known as cot death), including swaddling, dressing the infant too warmly, and other behaviors that may cause the sleeping child to overheat.^1^ In this issue of *EHP*, the authors of a new study of SIDS deaths report that hotter outdoor temperatures also may be a risk factor.^2^

SIDS deaths peak in infants between ages 2 and 4 months, and then decline,^3^ with males more likely to be affected than females.^4^ In 1994 the National Institute of Child Health and Human Development launched the Back to Sleep campaign (now known as the Safe to Sleep® campaign), which urged new parents to place their infants to sleep on their backs.^5^ Side- or stomach-sleeping can constrict a newborn’s airways, causing a dangerous drop in oxygen levels.^6^ (Stomach-sleeping may also interfere with a baby’s ability to regulate his body temperature, because a good deal of heat loss occurs through the face.^1^)

**Figure:**
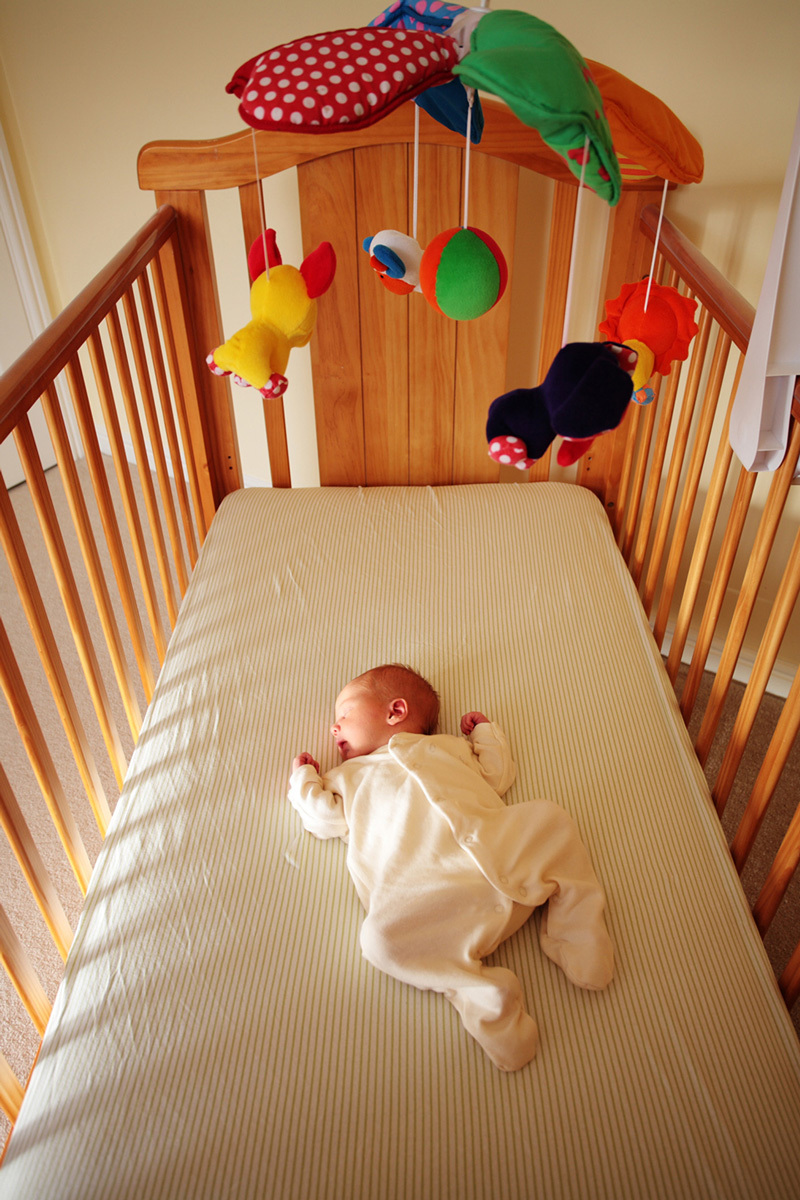
Overheating is one of the chief risk factors for SIDS. Parents should dress their babies warmly but not go overboard with clothing and blankets. © David Nigel Owens/Getty Images

The campaign was a tremendous success—in its first 10 years, U.S. SIDS rates decreased by more than 50%. However, rates have since plateaued,^7^ and an estimated 2,300 babies still die of SIDS each year in the United States.^8^

Although older children are able to rouse themselves and shift their sleeping position to regain adequate oxygen levels, these abilities are underdeveloped in very young infants.^9^ A baby’s thermoregulatory system also has not reached maturity. “Infants are not like adults; they don’t have the same ability to regulate temperature through sweating,” says Nathalie Auger, an epidemiologist at the Québec Public Health Institute and lead author of the new *EHP* study.

A 1993 study first linked infant thermoregulation and respiratory control to SIDS,^10^ and in 2008 scientists reported that the use of a fan in an infant’s bedroom appeared to help reduce SIDS risk.^11^ These studies, Auger believed, hinted that extreme heat events also might increase SIDS risk, although no one had actually tested that.

Auger and colleagues identified 196 certified cases of SIDS in metropolitan Montreal in April through October for the years 1981 through 2000. To analyze the effects of heat, the researchers used a case-crossover design, where each case of SIDS served as its own control. For each SIDS case, the researchers ascertained the maximum temperatures on the day of and the day before death. Then they compared these temperatures with the maximum temperatures of matched control days. Control days were chosen on the basis of each infant’s death date, such that for a child who died on a Saturday in July 2000, for instance, control days would consist of all the other Saturdays in that month. This study design allowed the researchers to control for confounders such as secondhand smoke exposure, birth weight, and sleep environment.

On the hottest days, when the temperature exceeded 29°C (84.2°F), infants had 2.78 times greater odds of dying from SIDS compared with days when the temperature was 20°C (68.0°F). The relationship between higher temperatures and SIDS was stronger for babies aged 3–12 months compared with those aged 1–2 months, with odds ratios of 3.90 and 1.73, respectively, for deaths on days with maximum temperatures of 29°C versus 20°C.^2^

“I have to congratulate the authors on doing SIDS research—it’s not an easy task,” says De-Kun Li, a senior research scientist at the Kaiser Permanente Northern California Division of Research, who was not involved in the study. However, he points out an important limitation of the study: Although the authors measured outdoor temperatures, they didn’t measure the actual temperature of the room in which the baby slept. Plenty of SIDS deaths occur in winter when babies are dressed too warmly, and the same could occur during heat waves when houses may be air-conditioned to the point of chilliness, he says.

“Understanding causes for SIDS remains a work in progress,” Li says. “It’s very hard to study.”
